# Thrombomodulin: a potential biomarker for sepsis-associated acute kidney injury resulting from bacterial infections

**DOI:** 10.3389/fcimb.2026.1710011

**Published:** 2026-06-01

**Authors:** Hao Hong, Mengya Xiao, Junyao Zheng, Yuting Wang, Jun Jin, Yang Xu, Ming Li

**Affiliations:** 1 Critical Care Medicine, The First Affiliated Hospital of Soochow University, Suzhou, China; 2 The First Affiliated Hospital of Soochow University, National Clinical Research Center for Hematologic Diseases, Jiangsu Institute of Hematology, Institute of Blood and Marrow Transplantation, Soochow University, Suzhou, China; 3 Laboratory Nephrology, The First Affiliated Hospital of Soochow University, Soochow, China

**Keywords:** acute kidney injury, bacterial infection, biomarker, sepsis, thrombomodulin

## Abstract

**Background:**

Sepsis-associated acute kidney injury (SA-AKI) is a common complication of sepsis. Serum creatinine is a delayed AKI marker influenced by multiple confounders and cannot reflect endothelial injury central to SA-AKI. Thrombomodulin (TM) is an endothelial biomarker linked to coagulation and inflammation, but its predictive value for SA-AKI in bacterial infection remains undefined. We aimed to evaluate the independent predictive performance of TM for SA-AKI in bacterial sepsis.

**Methods:**

This was a single-center retrospective observational study enrolling 81 patients with bacterial sepsis (37 with SA-AKI, 44 without SA-AKI) admitted to the intensive care unit of the First Affiliated Hospital of Soochow University. Blood samples were collected within 24 hours of sepsis diagnosis for TM measurement. For comparisons between two independent groups, Student’s t-test was used for normally distributed variables, while Mann–Whitney U test was utilized for non-normally distributed variables. For multi-group comparisons involving normal distribution with homogeneous variance data, One-way analysis of variance with Bonferroni *post-hoc* test was applied, whereas Mann–Whitney U test was utilized for non-normally distributed variables. Categorical variables were compared using the Chi-square test. Spearman’s correlation analysis was performed to assess relationships. Binary logistic regression analysis was conducted to evaluate the independent relationships with SA-AKI. A receiver operating characteristic (ROC) curve was employed to determine diagnostic accuracy. Subgroup analyses were performed stratified by vasoactive drug use and continuous renal replacement therapy (CRRT) status.

**Results:**

Thrombomodulin was significantly elevated in patients with AKI compared to that in non-AKI patients, and the odds ratio for thrombomodulin was 1.17 in a multivariable logistic regression analysis. The Area under curve (AUC) value for thrombomodulin in SA-AKI was 0.81. In subgroup analysis, thrombomodulin expression was significantly higher in the vasoactive drug group. The AUC of thrombomodulin with vasopressor use was 0.70. In the group that did not utilize vasoactive drugs, the AUC of thrombomodulin in SA-AKI was 0.81. Thrombomodulin expression was significantly higher in the Continuous Renal Replacement Therapy (CRRT) group. The AUC of Thrombomodulin (TM) when CRRT was used was 0.80. In the group that did not receive CRRT, the AUC of thrombomodulin predicted by SA-AKI was 0.75.

**Conclusions:**

Thrombomodulin may be valuable in independently predicting SA-AKI.

## 1 Introduction

Sepsis is a life-threatening organ dysfunction that results from an abnormal or dysregulated host response to infection. Sepsis-associated acute kidney injury (SA-AKI) significantly increases patient morbidity and mortality (Poston and Koyner, 2019). However, the pathological mechanism underlying SA-AKI remain unclear (Van der Slikke et al., 2021; Kuwabara et al., 2022). In clinical practice, a significant proportion of sepsis cases and sepsis-related acute kidney cases are associated with bacterial infections. Factors such as renal hypoperfusion, inflammation, coagulation disorders, microvascular dysfunction, endothelial damage, and tubular epithelial cell injury may contribute to the onset and progression of SA-AKI (Basalely and Menon, 2023; Zarbock et al., 2023a). However, serum creatinine, the gold standard for clinical diagnosis of AKI, has obvious limitations. It is a delayed indicator that only increases when renal function is significantly impaired, and is easily interfered by age, gender, muscle mass, hydration status and hemodynamic changes. Moreover, creatinine cannot reflect endothelial damage, coagulation disorders and inflammatory responses—the core pathological mechanisms of SA-AKI. Therefore, it is urgent to identify a novel, specific and early biomarker for SA-AKI secondary to bacterial sepsis.

Thrombomodulin (TM) is a single transmembrane, multidomain glycoprotein receptor primarily expressed by vascular endothelial cells, although it is also found in several other cell types, including neutrophils, synovial lining cells, monocytes, nucleated cells, and osteoblasts (Watanabe-Kusunoki et al., 2020). Initially, TM garnered significant attention from researchers because of its ability to regulate coagulation through thrombin activity (Giri et al., 2024). However, beyond its anticoagulant function, TM has also demonstrated anti-inflammatory properties (Iba and Levy, 2024). Recent studies have suggested that TM may regulate inflammation by interacting with HMGB1 and LPS-related antigens. Nevertheless, there remains a relative paucity of research on the role of TM in SA-AKI (Won et al., 2022; Kikuchi et al., 2024).

In this study, we aimed to investigate the predictive value of TM in SA-AKI resulting from bacterial infections. The progression of SA-AKI frequently necessitates the administration of vasoactive drugs and Continuous Renal Replacement Therapy (CRRT) to sustain circulation and enhance the internal environment as well as to address the progression of AKI. Consequently, we performed a subgroup analysis to determine whether TM retained its predictive value during the administration of vasoactive drugs and CRRT.

## 2 Methods

### 2.1 Study population

This single-center retrospective observational study was conducted in the intensive care unit of the First Affiliated Hospital of Suzhou University, Jiangsu Province, China, from May 2023 to May 2024. We estimated the sample size according to the Gpower software and set a 10% loss to follow-up rate. There were > 32 patients in each group. This study met the inclusion criteria for a minimum sample size. The inclusion criteria for this study were adults who met the diagnostic criteria for sepsis and SA-AKI (Zarbock et al., 2023b). Sepsis was defined in accordance with the third international consensus definition of sepsis and septic shock (Singer et al., 2016). AKI was defined according to the Kidney Disease Improving KDIGO criteria, utilizing daily serum creatinine measurements and hourly urine output data (Ostermann et al., 2020). In our study, we used the SA-AKI definition proposed by the ADQI 28 Working Group to guide our analysis (White et al., 2023). According to the ADQI 28 Working Group criteria, patients diagnosed with sepsis were divided into SA-AKI group (AKI occurred between day 1 and day 7 after sepsis diagnosis) and non-AKI group (no AKI occurred during sepsis hospitalization). However, it was determined that if AKI manifested before the diagnosis of sepsis, the patient would not qualify as having SA-AKI under the defined parameters. The exclusion criteria were as follows (1): individuals with inherent coagulation dysfunction (2), patients with severe liver disease (3), patients with severe immunodeficiency, and (4) pregnant or lactating individuals. The Research Ethics Committee of the First Affiliated Hospital of Soochow University approved this study protocol (no.757).

### 2.2 Laboratory measurements

To collect plasma, 3 ml of fasting venous blood was drawn early in the morning following admission and stored in EDTA anticoagulant tubes. The samples were maintained at room temperature for 20 min before centrifugation at 3000 rpm and 4 °C for 10 min. Subsequently, 500 µL of the upper plasma layer was transferred to a 2 ml cryovial and frozen at -80 °C. Biochemical and conventional hematology indicators were measured using an OLYMPUS AU2700 automatic biochemical analyzer (Olympus, Japan) and a Beckman LH750 automatic hematology analyzer (Beckman, USA). Blood coagulation was assessed using STA-R Evolution (STAGO, France). Cytokine levels were quantified by flow cytometry using a FACS Canto II (BD Biosciences, USA). Additionally, the HISCL-5000 (Sysmex, Germany) was employed to measure TM, Tissue - type Plasminogen Activator - Inhibitor 1 Complex t.

### 2.3 Statistical analysis

Statistical analysis was conducted using SPSS version 27.0, and GraphPad version 10.0 was employed for plotting. Data are presented as mean ± SD, median, and interquartile range (IQR) for normally and non-normally distributed data. For comparisons between two independent groups, Student’s t-test was used for normally distributed variables, while Mann–Whitney U test was utilized for non-normally distributed variables. For multi-group comparisons involving normal distribution with homogeneous variance data, One-way analysis of variance with Bonferroni *post-hoc* test was applied, whereas Mann–Whitney U test was utilized for non-normally distributed variables. Categorical variables were compared using the Chi-square test. Spearman’s correlation analysis was performed to assess relationships. Binary and ordered logistic regression analyses were conducted to evaluate the independent relationships with SA-AKI. For confounding factors, we adjusted for confounding bias by using multivariate logistic regression. The receiver operating characteristic (ROC) curve was used to determine the diagnostic accuracy, and the Youden index was calculated to establish the optimal cutoff point of the ROC curve. *P<* 0.05 (*) and *P<* 0.001 (**) were considered statistically significant.

## 3 Results

### 3.1 Study population

**Figure 1:**
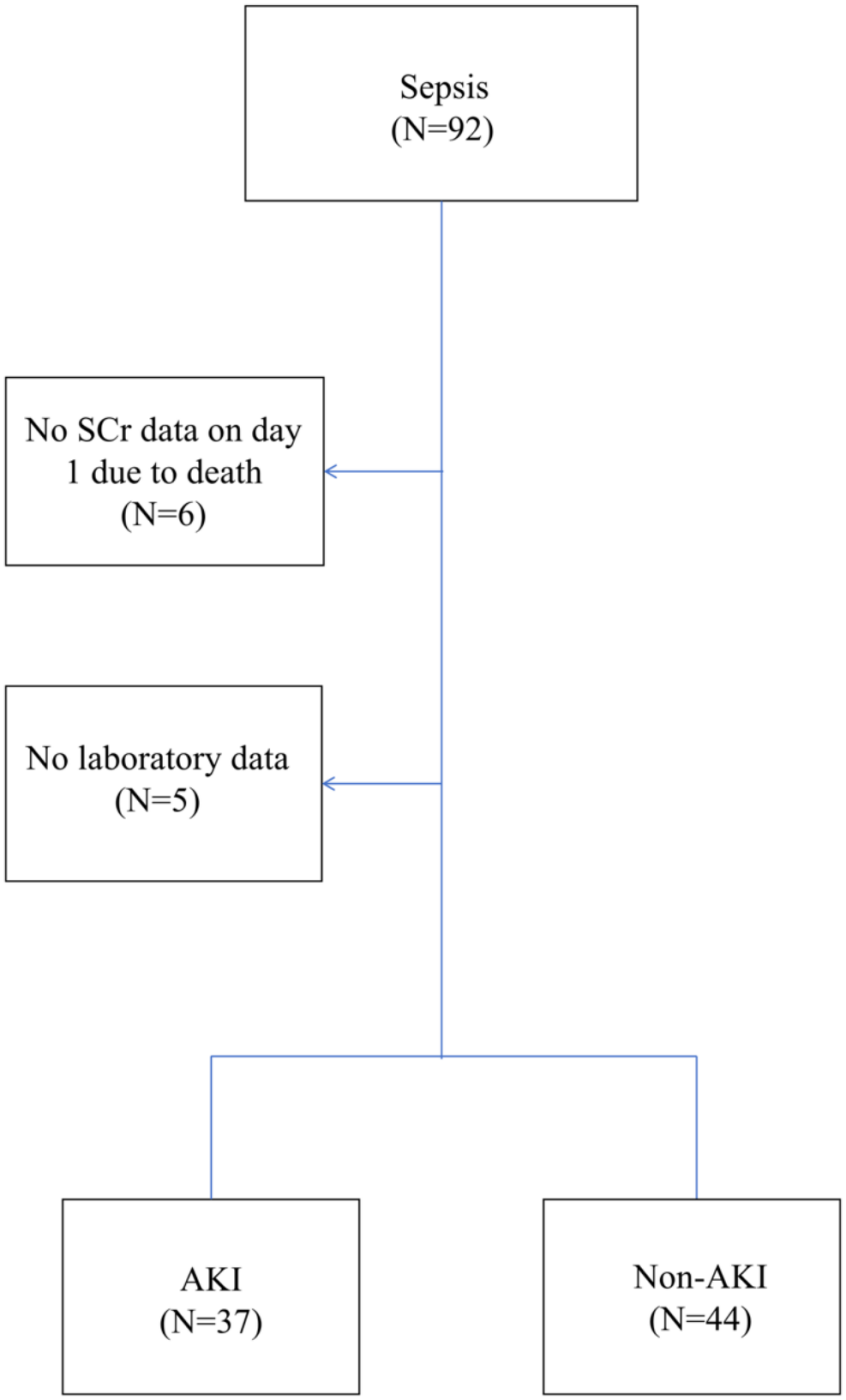
Inclusion and exclusion of patients. (SCr, serum creatinine).

During the study period, 92 patients with sepsis resulting from bacterial infections were included in the study. Eleven participants were excluded (1): five patients lacking laboratory data and (2) six patients who had no serum creatinine measurements due to death on day 1. Consequently, 81 sepsis patients were included in the study, consisting of 37 patients with AKI and 44 patients without AKI (**Figure 1**). There were no statistically significant differences in age, weight, height, and gender distribution between the sepsis group and the SA-AKI group. Specifically, the median age in the sepsis group was 75.00 (56.50, 80.00), and that in the SA-AKI group was 70.00 (54.00, 78.00); the median height in the sepsis group was 160.00 (160.00, 170.00), and that in the SA-AKI group was 170.00 (160.00, 172.00); the median weight in the sepsis group was 65.00 (58.00, 73.00), and that in the SA-AKI group was 65.00 (60.00, 70.00); the proportion of males in the sepsis group was 29.55% and that of females was 70.55%, while the proportion of males in the SA-AKI group was 24.32% and that of females was 75.78%. Compared to the non-AKI group, the AKI group exhibited significantly higher APACHE II scores (*P<*0.05), SOFA scores (*P<*0.05), and rates of CRRT (*P<*0.001). Additionally, vasoactive medication use was significantly more prevalent in the AKI group (*P <*0.05) (**Table 1**).

**Table 1:** Baseline characteristics of sepsis patients with and without AKI.

Variable	Sepsis (N = 44)	SA-AKI (N = 37)	*P* value
Age, years	75.00 (56.50, 80.00)	70.00 (54.00, 78.00)	0.61
Height, cm	166.00 (160.00, 170.00)	170.00 (160.00, 172.00)	0.58
Weight, kg	65.00 (58.00, 73.00)	65.00 (60.00, 70.00)	0.83
Male sex	29.55% (13/44)	24.32% (9/37)	0.42
APACHE II	15 (12, 20)	22 (16, 26)	< 0.05
SOFA	5 (4, 8)	9.5 (6, 12)	< 0.05
CKD	11.36% (5/44)	13.51% (5/37)	0.28
HT	63.64% (28/44)	62.16% (23/37)	0.73
DM	38.64% (17/44)	27.03% (10/37)	0.90
COPD	6.82% (3/44)	8.11% (3/37)	0.75
HF	9.1% (4/44)	13.6% (5/37)	0.82
Hepatic	9.09% (4/44)	8.11% (3/37)	0.98
Infection site			
Intracranial	2.27% (1/44)	2.70% (1/37)	
Thoracic	72.73% (32/44)	56.76% (21/37)	
Abdominal	9.09% (4/44)	27.03% (10/37)	
Urinary tract	11.36% (5/44)	10.81% (4/37)	
Soft tissue	6.82% (3/44)	5.41% (2/37)	
28-day mortality	6.82% (3/44)	18.92% (7/37)	0.10
CRRT	6.82% (3/44)	48.65% (18/37)	< 0.001
Vasopressors	27.27% (12/44)	56.76% (21/37)	0.03

AKI, Acute kidney injury; SA-AKI, sepsis-associated acute kidney injury; APACHE II Acute Physiology and Chronic Health Evaluation II score; SOFA Sequential Organ Failure Assessment; CKD Chronic kidney disease; HT, Hypertension; DM Diabetes mellitus; COPD Chronic obstructive pulmonary disease; HF Heart Failure; CRRT Continuous Renal Replacement Therapy. Student’s t-test was used for normally distributed variables, while Mann–Whitney U test was utilized for non-normally distributed variables.

### 3.2 Laboratory tests and endothelial biomarkers in the AKI and non-AKI groups

**Figure 2:**
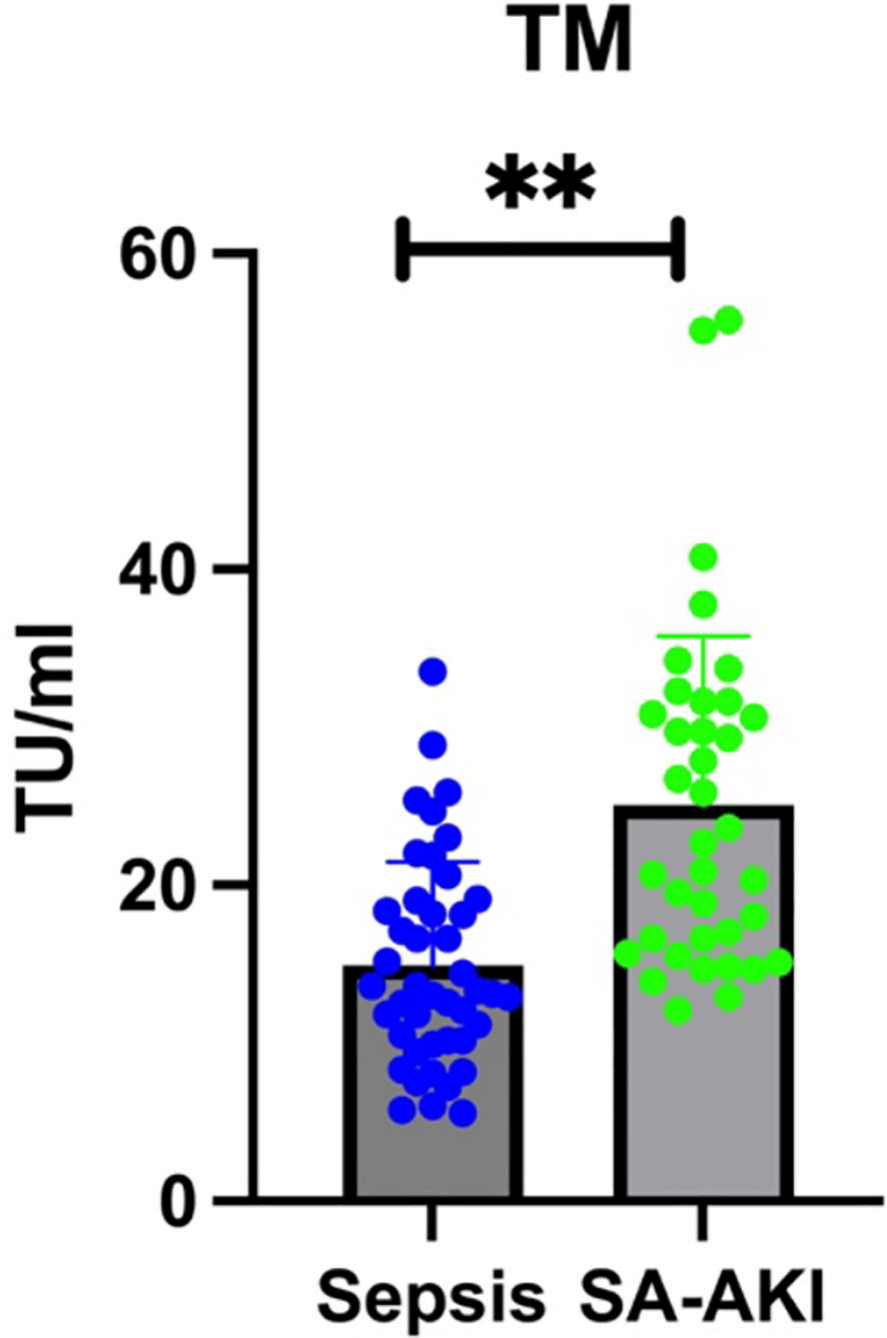
Thrombomodulin levels in patients with sepsis and SA-AKI. (TM, Thrombomodulin; SA-AKI, Sepsis-associated acute kidney injury). P < 0.001 (**) was considered statistically significant.

Among the endothelial biomarkers, TM (*P<*0.001) was significantly elevated in patients with AKI compared to that in non-AKI patients (**Figure 2**). In terms of blood coagulation indices, platelet count (PLT) (*P<*0.05) was significantly lower, whereas activated partial thromboplastin time (APTT) (*P<*0.001) was significantly higher in the AKI group. Interleukin-8 (IL-8) and interleukin-10 (IL-10) (*P<*0.05, *<*0.05 *P<*0.001, respectively) levels were significantly higher in the AKI group. Among the blood lipid indicators, triglyceride (TG) levels (*P<*0.05) were significantly elevated in the AKI group. Additionally, kidney-related indicators, including urea nitrogen (UREA) and cystatin C (CYSC) (*P<*0.001), were significantly higher. Among the internal environmental indicators, bicarbonate (HCO3-) and anion gap (ABE) (*P<*0.01, *<*0.05, respectively) were significantly lower (**Table 2**).

**Table 2:** Laboratory tests and endothelial biomarkers.

Variable	Sepsis (N = 44)	SA-AKI (N = 37)	*P* value
WBC (×10^9^/L)	6.42 (4.71, 20.60)	14.28 (9.18, 22.37)	0.44
RBC (×10^12^/L)	3.54 (3.32, 3.82)	3.58 (3.00, 3.91)	0.53
PLT (×10^9^/L)	204.00 (136.00, 295.00)	138.50 (72.25, 200.25)	0.03
CRP (mg/L)	153.69 (48.43, 238.38)	228.50 (97.99, 294.13)	0.10
TC (mmol/L)	3.09 (2.47, 3.63)	2.90 (2.23, 3.50)	0.31
TG (mmol/L)	1.25 (0.91, 1.80)	1.66 (1.39, 2.46)	0.01
SCR (μmol/L)	89.50 (61.70, 107.90)	224.40 (152.63, 362.33)	< 0.001
UREA (μmol/L)	9.60 (7.40, 14.40)	17.90 (12.85, 23.22)	< 0.001
CYSC (mg/L)	1.23 (0.83, 2.01)	1.88 (1.46, 3.23)	< 0.001
PH	7.43 (7.39, 7.47)	7.37 (7.26, 7.45)	< 0.05
HCO3- (mmol/L)	24.60 (22.40, 29.90)	17.70 (15.30, 21.98)	< 0.001
ABE	0.90 (-1.90, 5.30)	-5.40 (-9.18, -2.63)	< 0.05
PT (sec)	15.70 (15.00, 17.60)	16.55 (14.68, 20.30)	0.39
APTT (sec)	42.20 (35.20, 50.50)	48.85 (42.18, 61.03)	< 0.001
IL-6 (pg/ml)	28.93 (6.03, 118.95)	94.14 (22.62, 484.95)	0.06
IL-8 (pg/ml)	14.67 (1.93, 31.81)	37.25 (14.89, 106.46)	< 0.05
IL-10 (pg/ml)	1.90 (0.59, 7.14)	6.45 (3.01, 15.37)	< 0.001
TM (TU/ml)	11.80 (8.20, 17.10)	20.50 (14.78, 29.93)	< 0.001
t-PAIC (ng/ml)	9.97 (7.95, 11.99)	15.03 (11.33, 18.74)	0.06

WBC, White Blood Cell, RBC Red blood cell; PLT, platelet; CRP, C-reactive protein; TC, total cholesterol; TG, triglyceride; SCr, serum creatinine; UREA, Uric Acid, Cysc, cystatin C, ABE Actual surplus base; PT, Prothrombin time; APTT, activated partial thromboplastin time; TM, thrombomodulin; t-PAIC, Tissue Plasminogen Activator-Inhibitor Complex. Student’s t-test was used for normally distributed variables, while Mann–Whitney U test was utilized for non-normally distributed variables.

### 3.3 Correlation analysis

**Figure 3:**
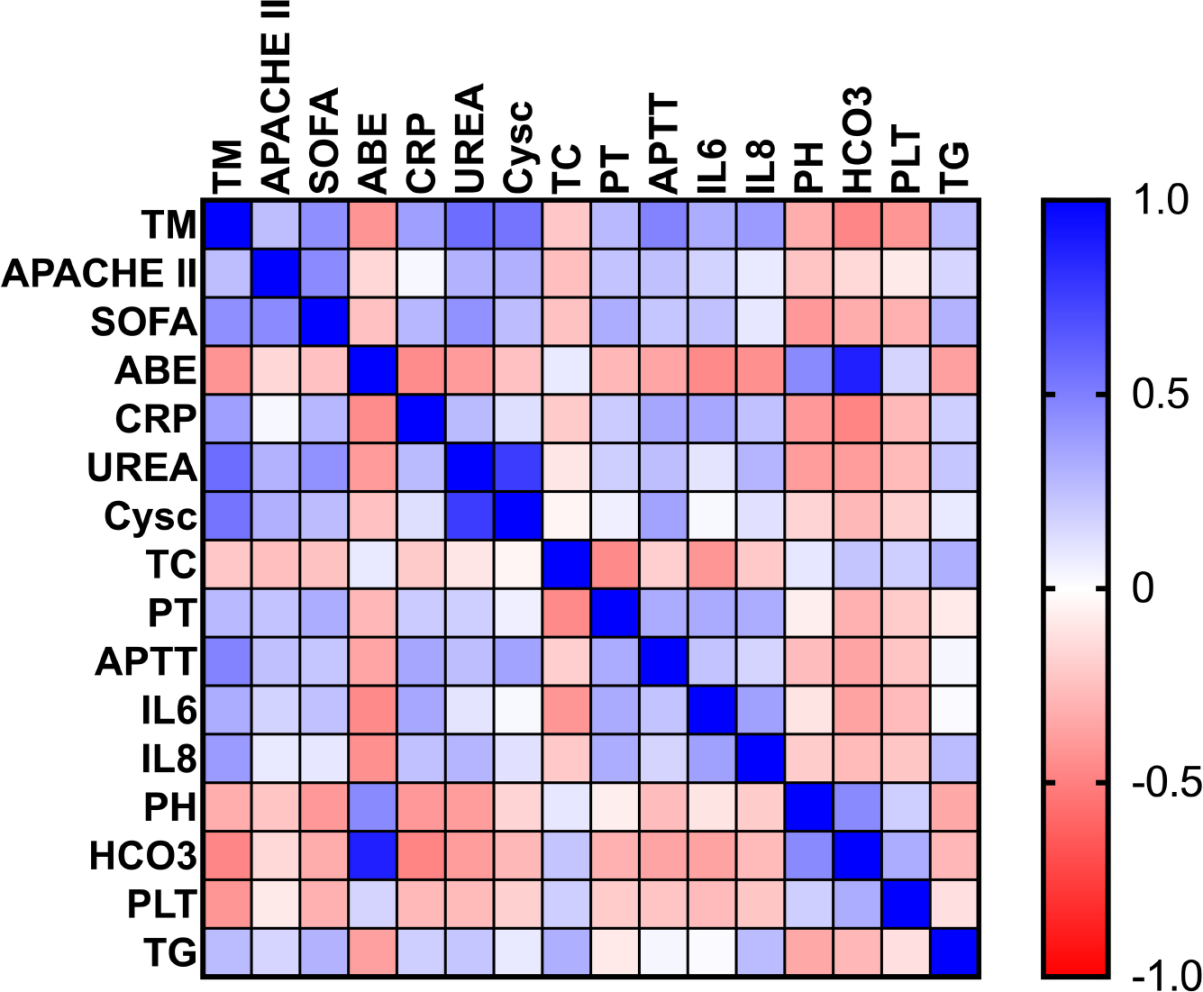
Correlation analysis. (TM, Thrombomodulin; ABE, anion gap; CRP, C-reactive protein; UREA, urea nitrogen; Cysc cystatin C; TC, total cholesterol; PT, Prothrombin time; APTT, activated partial thromboplastin time; IL-6, interleukin-6; IL-8, Interleukin-8; PLT, platelet count; TG, triglyceride).

Correlation analysis was performed to explore the relationships between TM and clinical, laboratory, and inflammatory parameters. The results showed that TM was positively correlated with multiple indicators reflecting disease severity, coagulation dysfunction, and inflammation (**Figure 3**):

Disease severity scores: APACHE II score (r=0.25, *P <*0.05) and SOFA score (r=0.44, *P* < 0.001), indicating that higher TM levels were associated with more severe organ dysfunction.

Coagulation indicators: APTT (r=0.49, *P* < 0.001), PT (r=0.27, *P <*0.05) and PLT (r=-0.41, *P* < 0.001), suggesting that TM elevation was linked to impaired endogenous coagulation and enhanced systemic inflammation.

Renal function indicators: UREA (r=0.57, *P* < 0.001) and CYSC (r=0.55, *P* < 0.001), reflecting a close association between TM and renal injury severity.

Inflammatory cytokines: CRP (r=0.38, *P* < 0.001), IL-8 (r=0.39, *P* < 0.001) and IL-6 (r=0.32, *P <*0.05), indicating that TM may be involved in the regulation of pro-inflammatory and anti-inflammatory responses in sepsis.

Metabolic indicators: TG (r=0.27, *P <*0.05), suggesting a potential interaction between TM and lipid metabolism in the pathophysiology of SA-AKI.

Conversely, TM was negatively correlated with indicators reflecting normal physiological function:

Blood gas and electrolyte indicators: PH (r=-0.32, *P <*0.05), ABE (r=-0.42, *P <*0.05) and HCO3- (r=-0.48, *P* < 0.001), indicating that TM elevation was associated with acid-base imbalance.

Coagulation and lipid indicators: TC (r=-0.22, *P <*0.05), suggesting that TM may be involved in platelet consumption and lipid metabolism disorders during sepsis.

### 3.4 Predictive value of TM in SA-AKI

**Figure 4:**
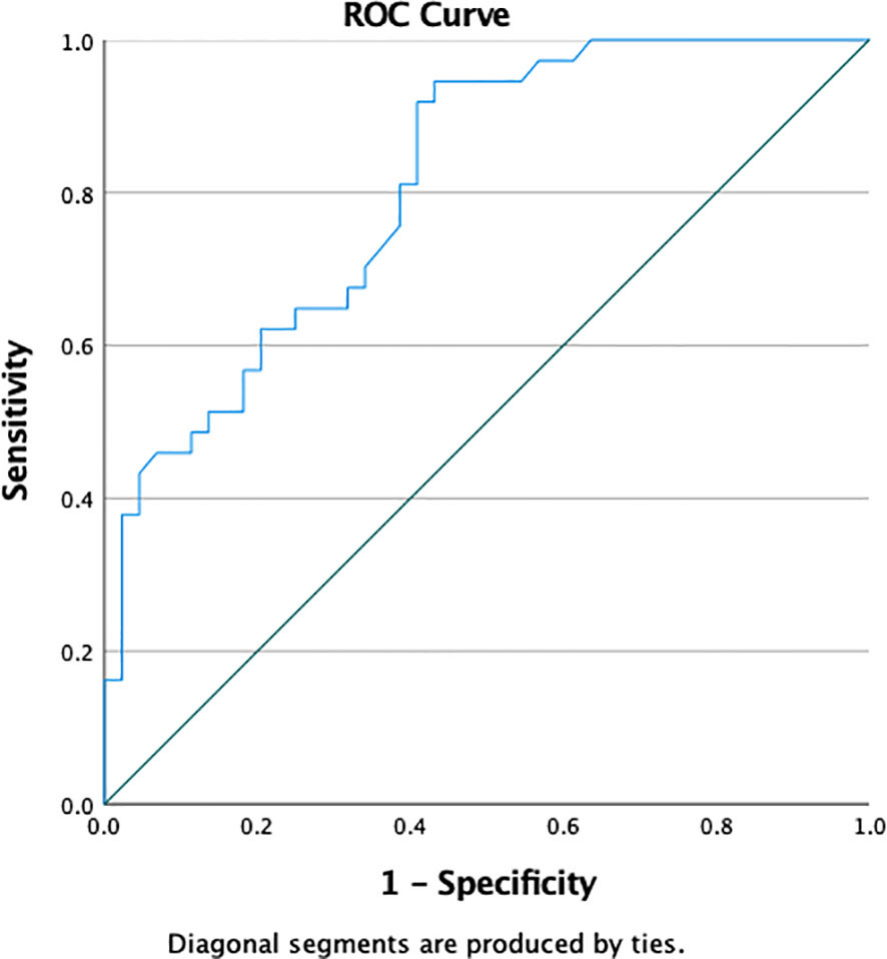
ROC curve of TM for SA-AKI. (TM, Thrombomodulin; SA-AKI, Sepsis-associated acute kidney injury).

To determine the predictive factors for AKI, we initially performed a single-factor logistic analysis, which revealed that the odds ratio (OR) for TM was 1.17 (*P<*0.001). Subsequently, based on the statistical differences observed between the two groups and the significant findings from the single-factor analysis, we included TM, IgG, pH, HCO3 −, ABE, APTT, TG, HDL, CYSC, urea, LMR, and PLT in a multivariable logistic regression analysis. The results indicated that TM was an independent predictor of AKI, with an OR of 1.15 (*P <*0.05) (**Table 3**). Furthermore, using area under the receiver operating characteristic curve analysis, the area under the curve (AUC) value for TM as a predictor of AKI was 0.81 (95%CI: 0.72-0.90, *P<*0.001), with a cut-off value of 0.51, a sensitivity of 0.95, and a specificity of 0.57 (**Figure 4**).

**Table 3:** Multivariate logistic regression analysis of SA-AKI.

Variable	B	S.E.	Wald	Df	Sig.	OR
sTM	0.14	0.05	6.62	1	0.01	1.15
ABE	-0.16	0.07	4.45	1	0.04	0.86
Constant	-2.86	1.04	7.59	1	0.01	0.06

SA-AKI Sepsis-associated acute kidney injury.

### 3.5 Subgroup analysis (vasopressor use)

**Figure 5:**
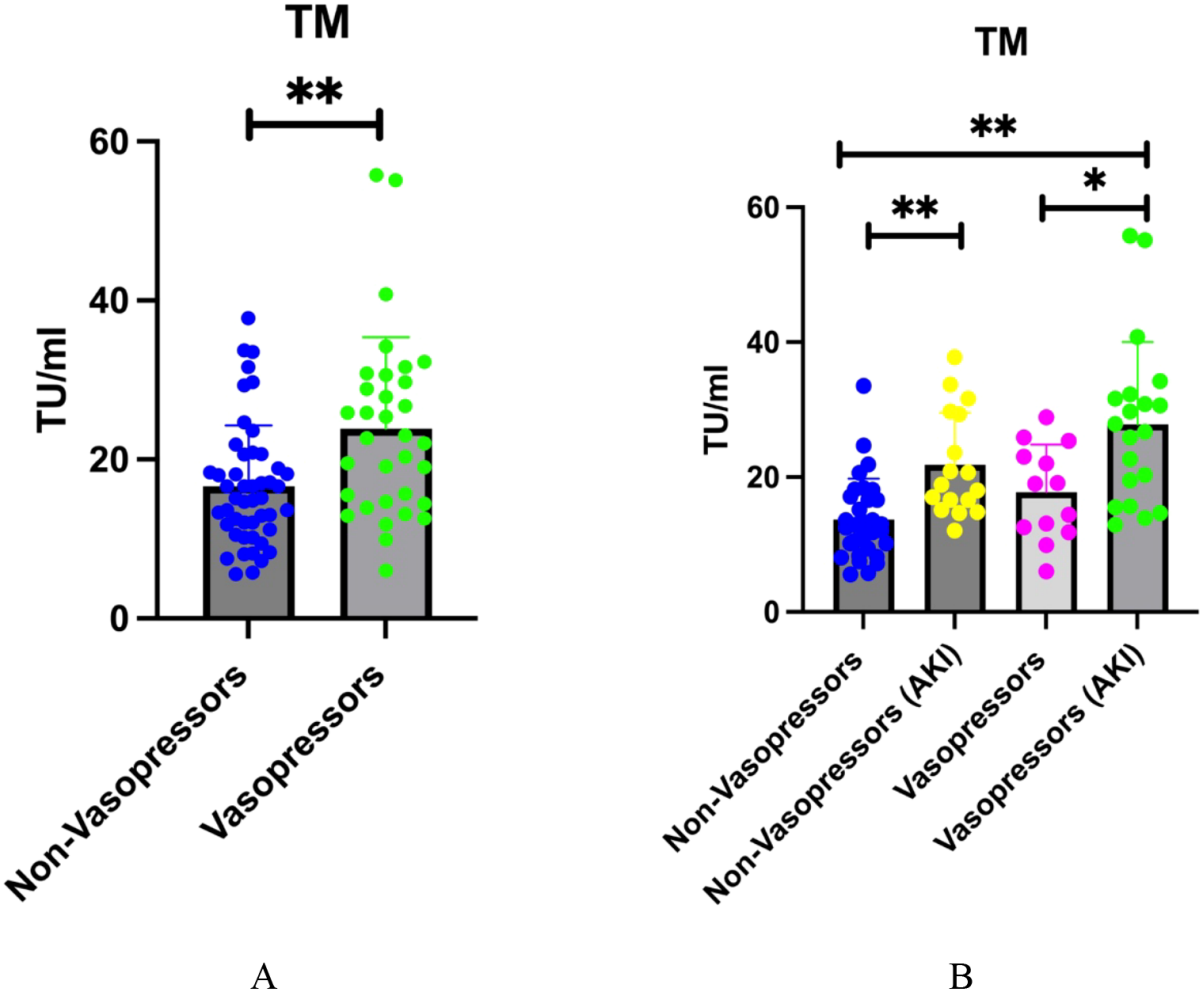
Thrombomodulin levels between patients with or without vasopressor medication **(A)**; intra-group analysis of thrombomodulin levels between sepsis and SA-AKI patients with or without vasopressor medication **(B)**. (SA-AKI, sepsis- associated acute kidney injury). P < 0.05 (*) and P < 0.001 (**) were considered statistically significant.

We investigated the expression in the presence or absence of vasopressor medication. Our findings indicate that TM expression was significantly higher in the vasoactive drug group (*P<*0.001) (**Figure 5A**). Further intra-group analysis revealed that in the group not receiving vasoactive drugs, TM expression in SA-AKI was significantly increased (*P<*0.001). In the group receiving vasoactive drugs, TM expression in SA-AKI also showed a significant increase (*P<*0.05) (**Figure 5B**).

**Figure 6:**
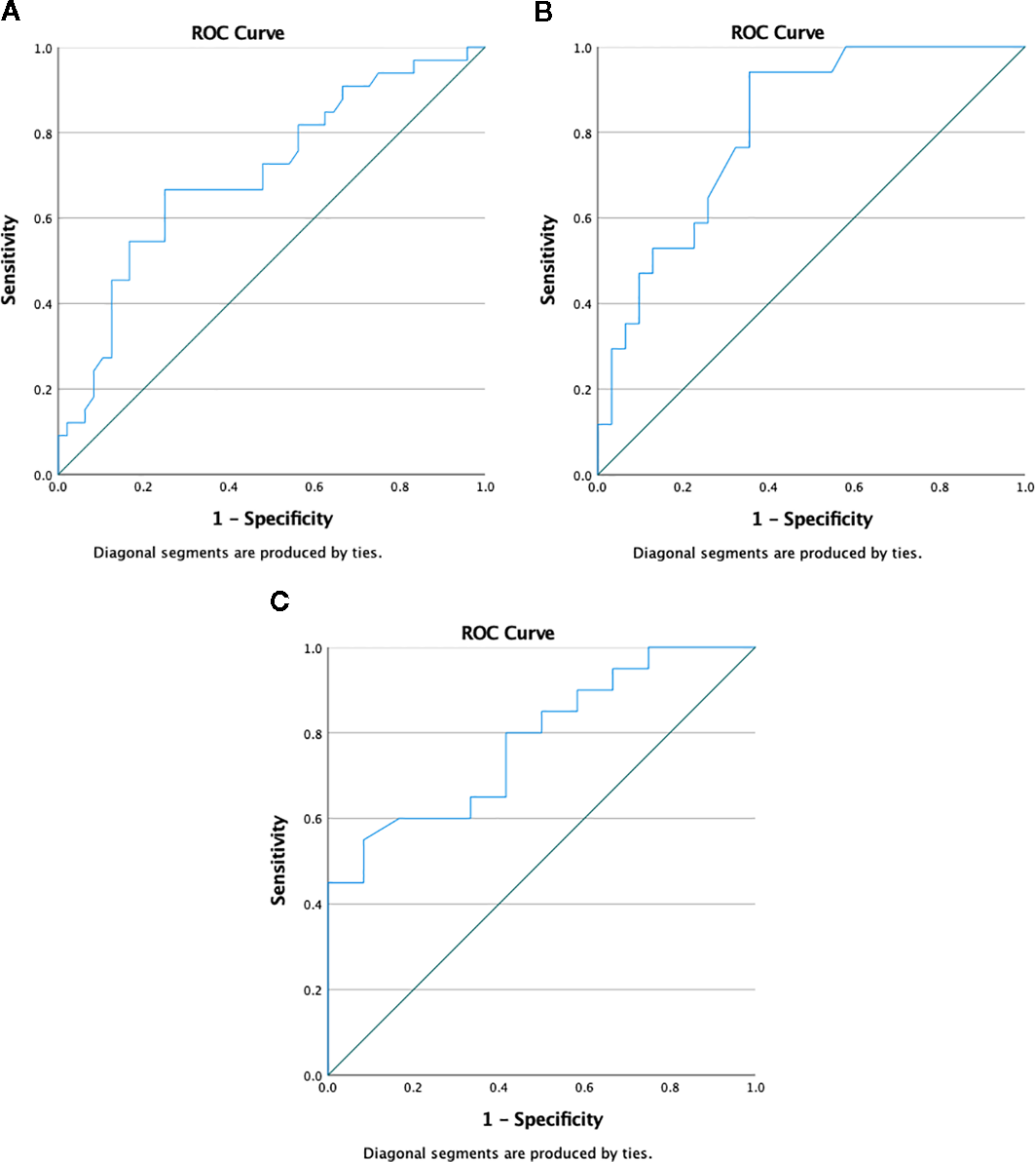
ROC curve of TM with the use of vasopressors **(A)** AUC of ROC predicted by TM. in SA-AKI without vasoactive drugs **(B)** AUC of ROC predicted by TM in SA-AKI by using vasoactive drugs. **(C)** (SA-AKI, Sepsis associated acute kidney injury) .

We generated the ROC curve and determined that the predicted AUC of ROC of TM with vasopressor use was 0.70 (95%CI: 0.59-0.82, *P <*0.05) (**Figure 6A**), with a cut-off value of 0.42, a sensitivity of 0.67, and a specificity of 0.75. Furthermore, intra-group predictive analyses were conducted. In the group not using vasoactive drugs, the AUC of ROC predicted by TM in SA-AKI was 0.81(95%CI: 0.69-0.93, *P<*0.001) (**Figure 6B**), with a cut-off value of 0.59, a sensitivity of 0.94, and a specificity of 0.65. In the group receiving vasoactive drugs, the AUC of ROC predicted by TM in SA-AKI was 0.78(95%CI: 0.62-0.94, *P <*0.05) and the cut-off value was 0.43, a sensitivity of 0.65, and a specificity of 0.83 (**Figure 6C**).

### 3.6 Subgroup analysis (CRRT)

**Figure 7:**
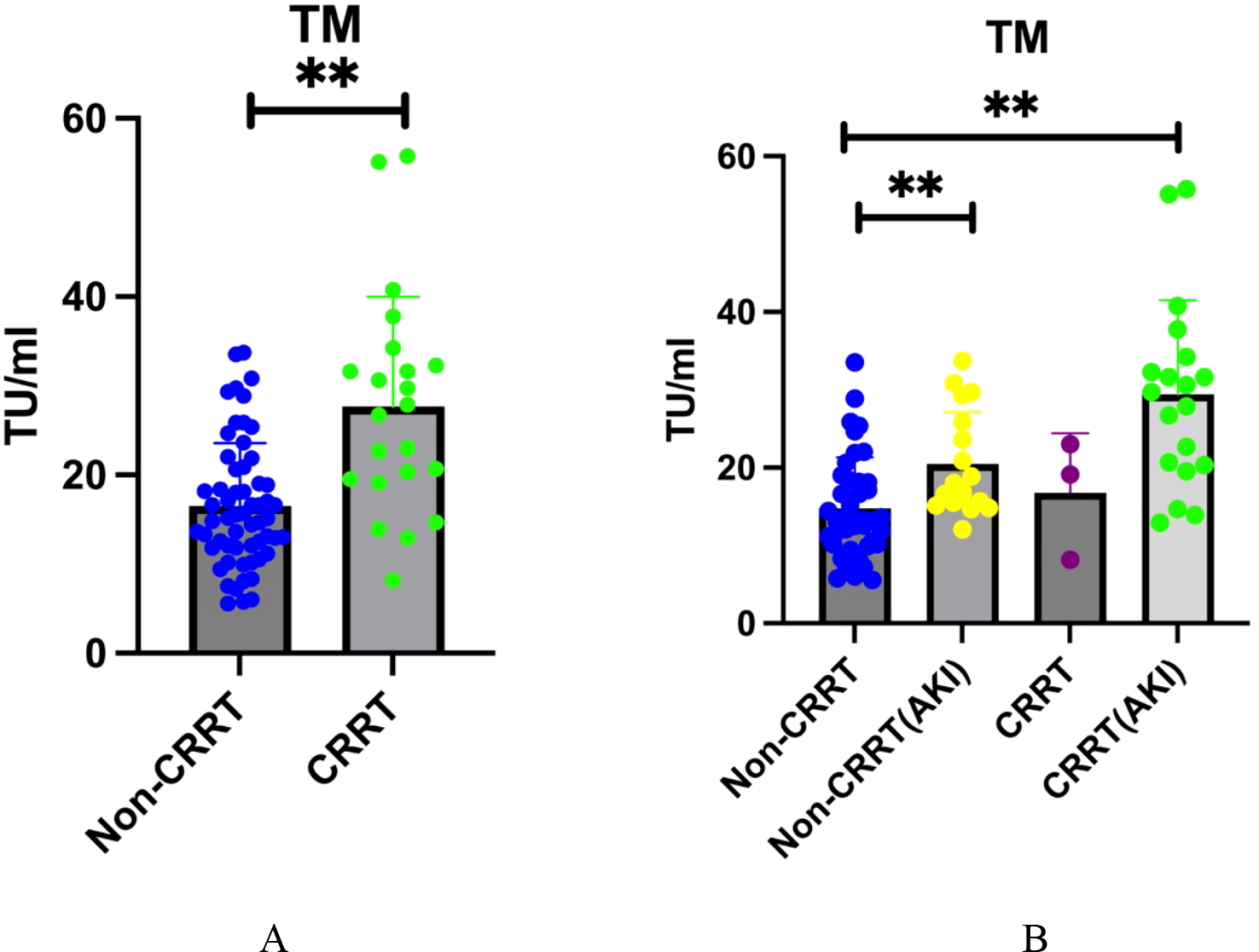
Thrombomodulin levels between patients with or without CRRT **(A)**; intra-group. Analysis of thrombomodulin levels in sepsis or SA-AKI patients with or without CRRT **(B)**. (SA-AKI, Sepsis-associated acute kidney injury; CRRT, Continuous Renal Replacement Therapy). P < 0.001 (**) was considered statistically significant.

We investigated the expression of TM with and absence of CRRT. Our findings indicate that TM expression was significantly higher in the CRRT group (*P<*0.001). Further intra-group analysis revealed that TM expression was significantly elevated in the non-CRRT group (*P<*0.001) (**Figure 7**).

**Figure 8:**
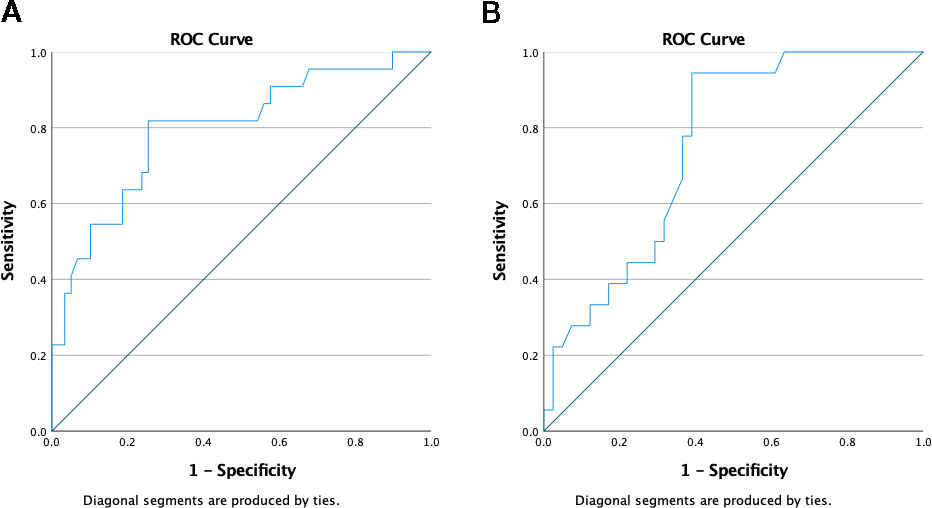
ROC curve of TM with the use of CRRT **(A)**: AUC of ROC predicted by TM in. SA-AKI without CRRT **(B)**. (SA-AKI, Sepsis-associated acute kidney injury; CRRT, Continuous Renal Replacement Therapy).

We constructed an ROC curve and determined that the predicted AUC of ROC of TM when CRRT was used was 0.80(95%CI: 0.68-0.91, *P<*0.001) (**Figure 8A**), with a cut-off value of 0.56, a sensitivity of 0.82 and a specificity of 0.75. Additional intra-group predictive analysis showed that in the group without CRRT, the AUC of ROC of TM predicted by SA-AKI was 0.75(95%CI: 0.63-0.88, *P <*0.05) (**Figure 8B**), with a cut-off value of 0.55, a sensitivity of 0.94, and a specificity of 0.61.

## 4 Discussion

In this study, we aimed to investigate the differential expression of TM in the SA-AKI and sepsis groups. Our findings indicate that TM is expressed at higher levels in SA-AKI patients than in sepsis patients. Through univariate and multivariate logistic regression analyses, we determined that TM was an independent risk factor for SA-AKI. Additionally, ROC curve analysis demonstrated that TM possesses good predictive performance for the occurrence of SA-AKI (AUC = 0.811, *P<*0.001), which aligns with the findings of several related studies (Katayama et al., 2017; Li et al., 2021; Nguyen et al., 2023; Pang et al., 2023). Furthermore, we conducted a subgroup analysis by regrouping the patients based on the use of vasoactive drugs. TM concentration in the group not using vasoactive drugs was lower than that in the group using vasoactive drugs. Further intergroup analysis revealed that TM levels were higher in the AKI group than in those without AKI. Interestingly, in another subgroup analysis, we found that TM concentration in patients undergoing CRRT was higher than that in the non-CRRT group. This observation may be attributed to the molecular mass of TM. Among the CRRT patients included in our statistical analysis, the predominant mode was continuous Veno venous hemodiafiltration (CVVHDF), which has a limited capacity for TM removal. Further intergroup analysis indicated that in the group without CRRT, TM levels were higher in the SA-AKI group than in the sepsis group.

Several studies have reported associations between endothelial dysfunction markers and the risk of SA-AKI in adults. Single-center studies have identified an independent association between TM and the risk of AKI in critically ill adults (Robinson-Cohen et al., 2016). Similar findings were observed in patients with sepsis enrolled in the multicenter Finnish Acute Kidney Injury (FINNAKI) cohort, where TM was linked to an independent risk of 90-day mortality (Inkinen et al., 2019). Furthermore, a cohort study involving children found that TM is independently associated with the risk of severe SA-AKI, leading to the establishment of a related prediction model (Atreya et al., 2023). Our study aligns with these previous findings. Notably, our subsequent subgroup analysis revealed that TM maintained a strong differential expression between groups that did and did not receive vasoactive drugs. The cohort of patients administered vasoactive drugs met two specific criteria: bedside ultrasound assessment confirmed the absence of cardiogenic shock and hypotension that remained uncorrected following fluid resuscitation for sepsis. The pathophysiological state of septic shock is characterized by disordered inflammatory immune responses, endothelial cell damage, and other contributing mechanisms. This finding suggests that patients with septic shock often exhibit more complex accumulation of TM. The AUC value of TM between the two groups was 0.70, indicating a certain level of predictive value. Consequently, we further analyzed the vasoactive drug group, in which TM was significantly differentially expressed between the sepsis and SA-AKI groups. The increased accumulation of TM in the SA-AKI group implies that clinicians may be able to monitor the occurrence of hypotension and implement corresponding intervention measures based on TM levels when treating patients with SA-AKI. AKI is associated with *in situ* cell damage, infiltration of inflammatory immune cells, thrombus formation, and other mechanisms (Gaut and Liapis, 2020; Liu et al., 2020; Pickkers et al., 2021). In the group not receiving vasoactive drugs, TM concentration was significantly higher in the AKI group than in the sepsis group. This finding suggests that TM may play a contributory role in sepsis complicated by AKI. However, the specific underlying mechanisms warrant further investigation. In an additional subgroup analysis, we observed that TM was differentially expressed between the non-CRRT and CRRT groups, demonstrating a strong predictive value (AUC = 0.80, *P<*0.001). Notably, the expression in the CRRT group was higher than that in the non-CRRT group. We hypothesized that this discrepancy may be attributed to the use of the CVVHDF mode in CRRT patients, which is characterized by a lower clearance efficiency for TM. Among the patients included in our study, those who did not undergo CRRT exhibited higher TM levels in the SA-AKI group than those in the sepsis group, suggesting a potential association with the occurrence of SA-AKI.

TM is a thrombin receptor that is expressed on the surface of endothelial cells. When thrombin binds to endothelial TM, protein C is activated, leading to the release of TM into the bloodstream, which subsequently inactivates the procoagulant function of thrombin (Ito et al., 2019). The interaction between TM and thrombin significantly diminishes the role of thrombin in the conversion of fibrinogen to fibrin and in the activation of coagulation factors V, VIII, and platelets (Vincent et al., 2019). TM reversibly disrupts the binding of thrombin to its procoagulation substrate. Evidence suggests that elevated levels of TM indicate endothelial damage and are associated with disseminated intravascular coagulation, multiple organ failure, and increased mortality (Lin et al., 2008). In this study, among the coagulation indicators we examined, TM, activated partial thromboplastin time APTT, and PLT were differentially expressed between the sepsis and SA-AKI groups, with TM identified as an independent risk factor distinguishing the two groups. Furthermore, correlation analysis revealed that TM was positively correlated with APTT (r=0.49, *P<*0.001) and negatively correlated with PLT (r=-0.41, *P<*0.001). Notably, one study indicated that in patients with cirrhosis, TM may provide a more accurate reflection of the coagulation status in this population than traditional indicators such as APTT (Zermatten et al., 2020). APTT reflects the endogenous coagulation pathway of the body. In this study, patients with severe acute kidney injury exhibited a longer APTT than those with sepsis did. The positive correlation between TM and APTT suggests a damage to the endogenous coagulation pathway. A multicenter study indicated that PLT count was reduced following recombinant thrombomodulin treatment (Kudo et al., 2021). Our findings revealed that the platelet levels in the SA-AKI patient group were significantly lower than those in the sepsis group. Furthermore, TM was negatively correlated with changes in PLT counts, indicating that the regulatory effect of TM on thrombus formation may be related to the modulation of PLT aggregation and subsequent consumption of thrombus. However, it is important to note that in patients with sepsis, platelet consumption is influenced by complex pathophysiological mechanisms that extend beyond the coagulation process.

Initially, TM was believed to exert its anti-inflammatory effects indirectly through the activation of activated protein C and inhibition of thrombin activity (Hirose et al., 2000; Feistritzer and Riewald, 2005). However, further studies have demonstrated that TM can mediate an anti-inflammatory response through two distinct mechanisms: first, by binding to the HMGB1 protein and second, by interacting with the carbohydrate Lewis Y antigen present on LPS from gram-negative bacteria (Altman et al., 2005; Dauphinee and Karsan, 2006; Ito et al., 2008; Shi et al., 2008). In relevant animal models of sepsis, pretreatment with recombinant TM protein, which encompasses all extracellular domains, significantly reduced mortality rates in LPS-induced sepsis (Nagato et al., 2009). Additionally, intrarenal injection of TM has been shown to ameliorate AKI resulting from inflammatory attacks (Ozaki et al., 2008; Sharfuddin et al., 2009). Our study found that the expression levels of TM, IL-8, IL-10, and LMR in the SA-AKI patient group were significantly higher than those in the sepsis group, with IL-8 showing a positive correlation with TM (r=0.39, *P<*0.001). Unfortunately, the other inflammatory factors examined did not demonstrate significant differences between the two groups. The mechanisms of indirect or direct interactions between TM and IL-8 in sepsis and SA-AKI disease models warrant further investigation.

Relevant studies have indicated that blood lipids may influence the development of sepsis through inflammatory responses (Gaskell et al., 2019; Chouchane et al., 2024). In this study, the concentration of triglycerides in patients with AKI was higher than that in the general sepsis group, demonstrating a weak positive correlation with TM (r=0.27, *P<*0.05). Notably, both TG and TM are involved in the binding of HMGB1 to regulate inflammatory responses. However, the interaction between TG and TM has not been reported previously.

This study has certain limitations. First, it was a single-center study. Second, we did not evaluate other biomarkers specifically related to AKI, such as urinary neutrophil gelatinase-associated lipocalin and kidney injury molecule-1 (Lumlertgul et al., 2020; Vandenberghe et al., 2020; Wu et al., 2023). Therefore, further multicenter studies with larger sample sizes are necessary to confirm the reliability of our findings.

## 5 Conclusions

Our study suggests that TM may have value in independently predicting sepsis-associated acute kidney injury, additional research is required to clarify the relationship between TM and the development of sepsis-associated kidney injury.

## Data Availability

Publicly available datasets were analyzed in this study. This data can be found here: the datasets used and/or analyzed during the current study are available from the corresponding author upon reasonable request.
